# Metanálisis sobre la utilidad de ELISA, PCR e inmunocromatografía en el diagnóstico de chikungunya

**DOI:** 10.26633/RPSP.2017.163

**Published:** 2017-12-05

**Authors:** Lizdany Flórez-Álvarez, Jaiberth Antonio Cardona-Arias

**Affiliations:** 1 Universidad de Antioquia Universidad de Antioquia Medellín Colombia Universidad de Antioquia, Medellín, Colombia. La correspondencia se debe dirigir a Jaiberth Antonio Cardona-Arias

**Keywords:** Virus Chikungunya, reacción en cadena de la polimerasa, ensayo de inmunoadsorción enzimática, inmunocromatografía, metanálisis, Chikungunya virus, polymerase chain reaction, enzyme-linked immunosorbent assay, immunochromatography, meta-analysis, Vírus Chikungunya, reação em cadeia da polimerase, ensaio de imunoadsorção enzimática, imunocromatografia, metanálise

## Abstract

**Objetivo.:**

Evaluar la utilidad de ELISA, PCR e inmunocromatografía para el diagnóstico de chikungunya.

**Métodos.:**

Se realizó un metanálisis de estudios que reportaran datos de validez diagnóstica, a partir de un protocolo ex-ante con seis estrategias de búsqueda en tres bases de datos multidisciplinarias. Se garantizó la reproducibilidad en la selección y extracción de información, se evaluó la calidad con la guía QUADAS (Quality Assessment of studies of Diagnostic Accuracy), los análisis se realizaron en MetaDisc con medidas puntuales, intervalos de confianza y resultados combinados bajo un modelo de efectos aleatorios.

**Resultados:**

Se incluyeron 19 estudios, uno con ELISA para anticuerpos IgG, dos con ELISA para antígenos, cinco con ELISA de anticuerpos IgM, ocho con qPCR y tres con inmunocromatografía. Los artículos fueron publicados entre 2009 y 2015, principalmente en India (37%), usando como prueba de referencia la combinación de sintomatología clínica, RT-PCR, ELISA, ensayo de neutralización o aislamiento viral. La población fue 1 108 individuos sanos, 394 con otra infección (principalmente dengue) y 1 288 con chikungunya. En ELISA para IgM y qPCR la sensibilidad y especificidad fueron mayores al 90%, el cociente de probabilidad positivo mayor a 10, el cociente de probabilidad negativo menor a 0,1; razón de Odds diagnóstica mayor a 100 y área bajo la curva de 0,99.

**Conclusión.:**

Se halló una excelente utilidad diagnóstica de la ELISA IgM y qPCR, mientras que para inmunocromatografía la utilidad fue escasa.

**Conclusion.:**

IgM ELISA and qPCR were found to be excellent for diagnosis, while immunochromatography was only of limited usefulness.

## Abstract (en)

**Objective.:**

Evaluate the usefulness of ELISA, PCR, and immunochromatography for the diagnosis of Chikungunya.

**Methods.:**

A meta-analysis of studies reporting diagnostic validity data was performed, using an ex-ante protocol with six search strategies in three multidisciplinary databases. Replicability in the selection and retrieval of information was guaranteed; quality was evaluated using the QUADAS (Quality Assessment of Diagnostic Accuracy Studies) guide; the analyses were performed in MetaDisc, with specific measures, confidence intervals, and combined results under a random-effects model.

**Results.:**

A total of 19 studies were included, one with IgG ELISA, two with antigencapture ELISA, five with IgM ELISA, eight with qPCR, and three with immunochromatography. The articles were published primarily in India (37%) between 2009 and 2015, using a combination of clinical symptoms, RT-PCR, ELISA, neutralization assay, or viral isolation as the reference test. The population consisted of 1 108 healthy individuals, 394 with another infection (mainly dengue), and 1 288 with Chikungunya. In IgM ELISA and qPCR, the sensitivity and specificity were greater than 90%, the positive probability quotient was greater than 10, the negative probability quotient was less than 0.1, the diagnostic odds ratio was greater than 100, and the area under the curve was 0.99.

## RESUMO

**Objetivo.:**

Avaliar a utilidade dos exames ELISA, PCR e imunocromatografia no diagnóstico de febre chikungunya.

**Métodos.:**

Uma meta-análise de estudos com dados de validade diagnóstica foi conduzida em três bases de dados multidisciplinares segundo um protocolo de avaliação ex-ante contendo seis estratégias de busca. Foi garantida a reprodutibilidade na seleção e extração de informação e avaliada a qualidade segundo os critérios do guia QUADAS (Quality Assessment of Studies of Diagnostic Accuracy). As análises foram realizadas no programa Meta-DiSc com estimativas pontuais, intervalos de confiança e resultados combinados em um modelo de efeitos aleatórios.

**Resultados.:**

A meta-análise incluiu 19 estudos, a saber: um sobre ELISA de anticorpos IgG, dois sobre ELISA de antígenos, cinco sobre ELISA de anticorpos IgM, oito sobre PCR quantitativa (qPCR) e três sobre imunocromatografia. Os artigos foram publicados entre 2009 e 2015, sobretudo na Índia (37%), e usaram como referência critérios clínicos, PCR em tempo real (RT-PCR), ELISA, ensaio de neutralização ou isolamento viral. A população abrangeu 1.108 indivíduos saudáveis, 394 que apresentavam outra infecção (mais comumente, dengue) e 1.288 com febre chikungunya. Com relação ao ELISA-IgM e qPCR, a sensibilidade e a especificidade foram superiores a 90%, a razão de probabilidade positiva foi maior que 10 e a razão de probabilidade negativa menor que 0,1, a razão de chances diagnóstica foi maior que 100 e a área sob a curva foi de 0,99.

**Conclusão.:**

Verificou-se excelente utilidade diagnóstica do ELISA-IgM e qPCR e baixa utilidade da imunocromatografia no diagnóstico de febre chikungunya.

Chikungunya o fiebre chikungunya es una arbovirosis causada por un Alphavirus de la familia *Togaviridae.* El virus, que recibe el mismo nombre (CHIKV) es transmitido por la picadura de mosquitos distribuidos en regiones tropicales y subtropicales, incluyendo *Aedes aegypti* y *Aedes albopictus* que son vectores de otros virus como dengue, virus del Nilo occidental y Zika, los cuales generan cuadros clínicos similares (1,2).

Esta enfermedad se presenta de manera aguda, y se caracteriza por la triada de fiebre, rash y artralgia; puede ser incapacitante y persistir por semanas o meses después de la fase aguda de la infección (3). La presencia del virus estaba limitada a África y algunas regiones del sur de Asia hasta la epidemia de la isla de la Reunión en 2005 que afectó a más de 300 000 personas (4). En diciembre de 2013 se reportó la circulación de CHIKV en el Caribe (5) y en Junio de 2015 la Organización Mundial de la Salud reportó más de un millón de casos sospechosos y alrededor de 30 000 casos confirmados de chikungunya en América (5,6), haciendo de esta arbovirosis un problema de salud pública en el continente (6).

Ante la ausencia de una vacuna o tratamiento específico, el diagnóstico oportuno de chikungunya es importante para monitorear y controlar su expansión (7), particularmente en regiones que aún no han sido afectadas por la epidemia y donde se presenta circulación simultánea de otras arbovirosis como dengue y zika. Generalmente el diagnóstico se realiza con base en la sintomatología del paciente y se confirma a través del laboratorio; esto evidencia la necesidad de evaluar la utilidad de las pruebas disponibles como base de la detección precoz y el direccionamiento del tratamiento.

Varios estudios han evaluado la utilidad de las pruebas diagnósticas reportando sensibilidad para ELISA de anticuerpos IgM entre 91% (8) y 100% (9);y especificidad entre 81% (8) y 100% (9) y valores predictivos positivos entre 67%(8) y 100% (9), evidenciando heterogeneidad en los parámetros de validez, la cual es extrapolable a otras pruebas como ELISA de antígenos, inmunocromatografía IgM y qPCR, a esto se suman otras limitaciones relacionadas con el bajo número de pacientes evaluados en estos estudios; y el hecho que algunos no incluyen control con grupos sanos, o presentan un reporte incompleto de los parámetros de evaluación de la utilidad diagnóstica para cada prueba, en la medida que no incluyen los cocientes de probabilidad, razones de verosimilitud, ni la razón de Odds diagnóstica.

Las limitaciones expuestas se subsanan con el desarrollo de una revisión sistemática, la cual mejora las posibilidades de extrapolación de los resultados, mejora la validez externa de las conclusiones, aumenta la precisión estadística de los análisis y expone todos los parámetros requeridos para evaluar la utilidad diagnóstica de las pruebas identificadas. Por ello, el objetivo de este estudio fue evaluar la utilidad de ELISA, PCR e inmunocromatografía para el diagnóstico de chikungunya, a partir de estudios publicados en la literatura científica mundial.

## MATERIALES Y MÉTODOS

Se realizó una revisión sistemática de la literatura con metanálisis, siguiendo la siguiente metodología:

### Protocolo de búsqueda y selección de los estudios según la guía PRISMA (Preferred Reporting Items for Systematic Reviews and Meta-Analyses)

Para la identificación o búsqueda de información se realizó una búsqueda de artículos originales publicados en Pubmed, Science Direct, y Embase, utilizando los términos virus Chikungunya y el nombre de las pruebas diagnósticas. El nombre del virus se combinó con las pruebas diagnósticas a través del booleano AND, con lo cual resultaron seis estrategias de búsqueda: Chikungunya virus AND Detection, Chikungunya virus AND Diagnosis, Chikungunya virus AND PCR, Chikungunya virus AND ELISA, Chikungunya virus AND Rapid test y Chikungunya virus AND Isolation. En la búsqueda no se realizó restricción por año de publicación ni idioma.

Algunas sintaxis utilizadas en las búsquedas fueron: (Chikungunya virus [Title/Abstract]) AND Detection[Title/Abstract], TITLE-ABSTR-KEY(Chikungunya virus) and TITLE-ABSTR-KEY(Diagnosis), (ab:(Chikungunya virus AND PCR)), (Chikungunya virus[Title/Abstract]) AND PCR[Title/Abstract].

Se realizó la búsqueda por sensibilidad (sin restringirla a términos DeCS o MeSH) con el fin de obtener el mayor número de estudios posibles en las bases de datos, aplicando los filtros de cada una de ellas con el fin de disminuir la cantidad de artículos que se podría importar desde el programa EndNote Web, facilitar la eliminación de duplicados y mejorar la reproducibilidad.

En la fase tamización se aplicaron los criterios de inclusión de un estudio original, con términos de búsqueda en título, resumen o palabra clave; se incluyeron investigaciones de evaluación diagnóstica desarrollados en humanos, que reportaran la sensibilidad y especificidad de las pruebas en evaluación, y que especificaran el protocolo de ejecución de las técnicas diagnósticas; en esta etapa se realizó la eliminación de los artículos duplicados exportando los resultados a una fuente común en EndNote Web.

Para la elección o aplicación de los criterios de exclusión, se descartaron artículos que no hubiesen realizado cegamiento entre evaluaciones, que no definieron la prueba o criterio de referencia para discriminar sanos de infectados, aplicación de las técnicas diagnósticas en otras virosis como el zika y el dengue; manuscritos con bajos tamaños de muestra (casos o series hasta de nueve casos) y artículos con información incompleta o que no especificaron perdidas de información y/o pacientes en el desarrollo del estudio.

Los estudios que cumplían los criterios de inclusión fueron leídos en su totalidad para aplicar los criterios de exclusión; posterior a ello se determinó el número de investigaciones que se incluirían en la síntesis cuantitativa y en el metanálisis.

### Recolección de la información

Las variables a analizar en cada estudio fueron el país de estudio, año de publicación, técnica evaluada, prueba de referencia empleada, sujetos estudiados clasificados en: verdaderos positivos (individuos con la infección que presentaban un resultado positivo), falsos positivos (individuos sin la infección que presentaban un resultado positivo), falsos negativos (individuos con la infección que presentaban un resultado negativo), verdaderos negativos (individuos sin la infección que presentaban un resultado negativo), y tipo de muestra. Para la extracción de la información se diseñó una hoja de cálculo en formato Excel.

### Análisis de reproducibilidad y evaluación de la calidad de los artículos

*A priori* se determinó que la reproducibilidad de la búsqueda y selección de los estudios se haría por consenso, mientras que la reproducibilidad de la extracción se hizo con el diligenciamiento independiente de los datos en Excel por parte de dos investigadores. La calidad metodológica de los estudios se hizo con los 14 ítems de la guía QUADAS (*Quality Assessment of studies of Diagnostic Accuracy*) y al finalizar esta fase se determinó el riesgo de sesgos en la selección de los individuos, en la prueba de referencia, en la prueba evaluada y en el tiempo de realización de las pruebas.

### Análisis de la información

Para cada artículo se calculó sensibilidad, especificidad, razones de verosimilitud o cocientes de probabilidad (CP), Razón de Odds Diagnóstica (ORD) y curva ROC, con sus intervalos de confianza del 95%. Los CP se categorizaron de la siguiente manera: a) excelente ayuda en el diagnóstico: CPN <0,1 y CPP >10; b) buena ayuda diagnóstica o de importancia clínica: CPN entre 0,1-0,2 y CPP 5-10; c) poca ayuda al clínico: CPN 0,21-0,50 y CPP 2,1-4,9; y d) la prueba no presentaba capacidad discriminante para CPN 0,51-1,0 o CPP 1,0-2,0. La ORD se categorizó en regular para resultados cercanos a 1,0 y excelente para valores mayores a 100. El área bajo la curva se tomó como excelente ayuda diagnóstica para valores cercanos a 100. Todos los análisis se realizaron bajo modelos de efectos aleatorios en el software *Meta-analysis of studies of evaluations of Diagnostic and Screening tests Meta-DiSs* con una significación del 0,05.

Para la estimación de las medidas combinadas se utilizó la Prueba Q (χ2) de Der Simonian-Laird, se agregó 0,5 a las celdas con valor de cero, los intervalos de confianza del 95% se corrigieron con una estimación por sobre-dispersión. estudio individual sobre el resultado global.

## RESULTADOS

**FIGURA 1.**
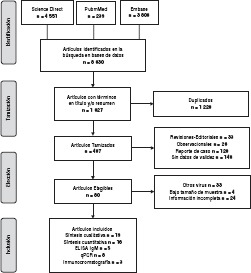
Flujograma de selección de los estudios

En la búsqueda inicial se identificaron 8 630 estudios de los cuales 1 627 incluían los términos de búsqueda en título o resumen; posterior a la eliminación de duplicados se tamizaron 407 manuscritos con lectura del resumen y la metodología, de éstos 33 correspondían a estudios secundarios, 26 eran descriptivos o analíticos, 120 reportes de casos o series de casos y en 148 no se incluyó la descripción de la sensibilidad y/o especificidad de las pruebas diagnósticas. Se realizó lectura completa de 80 estudios elegibles, de los cuales 33 evaluaron pruebas diagnósticas en otras infecciones virales, 4 incluyeron menos de diez pacientes y 24 presentaron información incompleta o no especificaron pérdidas de pacientes; con lo cual se incluyeron 19 manuscritos en la síntesis cualitativa, uno con ELISA para anticuerpos tipo IgG, dos con ELISA para antígenos, cinco con ELISA de anticuerpos tipo IgM, ocho con qPCR (2 cuantitativas y 6 cualitativas) y tres con inmunocromatografía; la síntesis cuantitativa sólo pudo realizarse para las tres últimas pruebas (figura 1).

 Los artículos fueron publicados desde 2009 hasta 2015, principalmente en India (n=7), usando como prueba de referencia el criterio clínico sumado al resultado de pruebas como RT-PCR, ELISA, Ensayo de neutralización y aislamiento viral. La población fue 2 790 individuos distribuidos en 1 108 sanos, 394 con otra infección (principalmente dengue) y 1 288 con chikungunya. Algunos estudios incluyeron dos subgrupos de análisis; por ejemplo, el grupo de Wasonga comparó ELISA IgM contra FRNT y frente a una ELISA IgM adicional; mientras que el grupo de Kosasih comparó Inmunocromatografía Onsite e Inmunocromatografía Biolab contra Elisa para IgM (cuadro 1).

En todos los estudios se usó muestra de suero, con excepción del estudio de Andriamandimby y col. (10) en el cual se usó sangre total; en el estudio de Shukla y cols. (11) además del suero se usó LCR; en la mayoría de los artículos que usaron RT-PCR para detección de CHIKV se amplificaron fragmentos del gen E1 y NS3.

**FIGURA 2.**
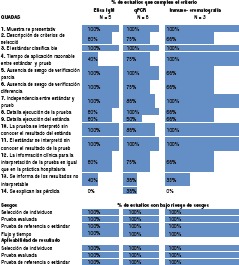
Evaluación de la calidad metodológica de los estudios y riesgo de sesgos

Todos los estudios incluidos en el metanálisis de las pruebas ELISA para anticuerpos IgM, qPCR e inmunocromatografia IgM presentaron bajo riesgo de sesgos en la selección de los sujetos de estudio y las pruebas comparadas; en los criterios de calidad metodológica muchos estudios no hacen explícitos los tiempos de aplicación del estándar y la prueba evaluada, o detalles operativos de las pruebas, sin embargo, estas características pudieron inferirse en algunos protocolos referenciados en los manuscritos (figura 2).

**FIGURE 3.**
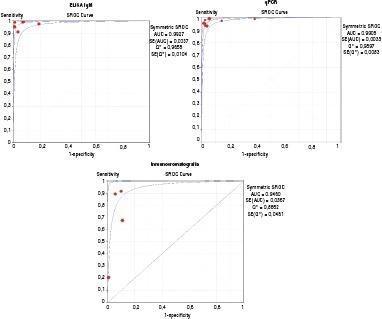
Área Bajo la Curva de los estudios que evaluaron ELISA IgM, qPCR e inmunocromatografía

En el cuadro 2 se observan los valores de utilidad diagnóstica para cada uno de los estudios incluidos en el metanálisis, así como la medida combinada para cada una de las tres pruebas, la evaluación de homogeneidad y el grado de correlación entre las pruebas y el criterio de referencia; destacándose el excelente desempeño diagnóstico de la ELISA IgM y la qPCR en la medida que presentaron sensibilidad y especificidad mayor al 90%, CPP mayor a 10, CPN menor a 0,1 y ORD mayor a 100, a diferencia de lo hallado para la Inmunocromatografía (cuadro 2). De forma global, el área bajo la curva fue de 0,993 para ELISA IgM, 0,990 en qPCR y 0,946 para la inmunocromatografìa (figura 3). En la inmunocromatografía las estimaciones globales se basan en la combinación de cuatro grupos (tres investigaciones) que incluyeron 354 individuos con CHIKV y 270 con otras infecciones; en éstos se halló una alta variabilidad para la sensibilidad (no así para los demás parámetros de utilidad diagnóstica) tanto intra como inter-estudios, lo que implica un bajo poder estadístico para comparar esta estimación con la hallada para las demás pruebas.

En el análisis de sensibilidad de los estudios que evaluaron ELISA IgM, para el CPP el porcentaje de peso de los estudios osciló entre 10,6% (9) y 18,4%; en el CPN entre 7,0% (9,10,13) y 12,6% (8), para la ORD fluctuó entre 6,7% (9) y 2,8% (8) lo que evidencia que ninguno de los estudios presentó una mayor influencia sobre la medida combinada. Hallazgos similares se registraron para qPCR con porcentaje de peso entre 7,2% (20)y 16,2% para CPP, 4,0% (13) y 16,6% para CPN y 4,5% a 18,0% (20) para la ORD.

**Fuente: **elaboración propia a partir de los resultados presentados.

En el análisis de subgrupos o meta-regresión, se realizó la estimación de las medidas de utilidad diagnóstica para ELISA IgM, excluyendo los estudios de Wasonga y cols. (8) que emplearon un estándar diferente a los demás; en este nuevo análisis se halló Correlación de Spearman = 1,000 (p=0,000); sensibilidad de 98,4% (IC95%=95,9-99,6) con heterogeneidad entre los estudios (I2=65,5%p=0,0034); especificidad de 97,6%(IC95%=94,8-99,1) con homogeneidad-entre los estudios (I2=57,2% p=0,072);CPP 27,3 (IC95%=8,8-84,8) con homoge-neidad entre los estudios (I2=49,0%p=0,117); CPN 0,03 (IC95%=0,01-0,07)con homogeneidad entre los estudios(I2=1,7% p=0,384); ORD 2286 (IC95%=539-9688) con homogeneidad entre losestudios (I2=0,0% p=0,909) y Área bajo lacurva 0,997; parámetros estadísticamentesimilares a los hallados para la totalidad de estudios.

Al repetir los análisis para qPCR excluyendo el estudio de Edwards y cols. (14) que utilizó un estándar diferente a losdemás, se halló Correlación de Spearman=0,408 (p=0,364); sensibilidad de 97,0% (IC95%=94,3-98,6) con homogeneidad-entre los estudios (I2=45,3% p=0,090); especificidad de 95,6% (IC95%=93,6-97,2) con heterogeneidad entre los estudios74 (I2=77,8% p=0,000); CPP 20,2 (IC95%=6,5-62,4) con heterogeneidad entre los estudios (I2=85,5% p=0,000); CPN 0,05 (IC95%=0,03-0,09) con homogeneidad-entre los estudios (I2=0,0% p=0,692); ORD 609 (IC95%=253-1468) con homo-geneidad entre los estudios (I2=0,0%p=0,773) y Área bajo la curva 0,991.

**CUADRO 1.** Caracterización de los estudios según año y país de publicación, prueba de referencia y número de sujetos estudiados

Autor (referencia)	Año	País	Prueba referencia	# Sujetos
					Sanos	Enfermos
ELISA IgM					
Shukla J (11)	2009	India	RT-PCR	117	84
Bhatnagar S (9)	2014	India	RT-PCR/IgM	45^a^	45
Matheus S (12)	2015	Guyana	RT-PCR/IgM	65	56
Goh L (13)	2015	Australia	RT-PCR	20^b^b	60
Wasonga C (8)	2015	Kenia	FRNT	92	56
Wasonga C (8)	2015	Kenia	ELISA para IgM	107	41
qPCR					
Edwards C (14)	2007	Inglaterra	ELISA para IgG	37	18
Santhosh S (15)	2007	India	Aislamiento/IgM/RT-PCR	10	51
Kumar Dash P (16)	2008	India	RT-PCR y ELISA para IgM	20	22
Pongsiri P (17)	2012	Tailandia	RT-PCR	230^a^	60
Andriamandimby S (10)	2013	Madagascar	RT-PCR	108	73
Chiam C (18)	2013	Malasia Aislamiento / PCR / IFI(IgM) / Seroconversion	20	30
Chen H (19)	2013	Singapur	RT-PCR	20	22
Chen H (20)	2015	Singapur	RT-PCRe	136^a^	47
Inmunocromatografia IgM					
Arya S (21)	2011	India	ELISA para IgM	86	14
Kosasih H (22)	2012	Indonesia	ELISA para IgM	74^b^	132
Kosasih H (22)	2012	Indonesia	ELISA para IgM	74^b^	132
Okabayasi T (23)	2014	Tailandia	RT-PCR	36	76
Otras					
ELISA (IgG)				
Grivard P (4)	2007	Francia	ELISA para IgG/IgM	97	46
ELISA Ag				
Kashyap R (24)	2009	India	ELISA IgM	19	119
Yathi K (25)	2013	India	Aislamiento viral / PCR	89^a^	104

***Fuente: elaboración propia a partir de los resultados presentados*.**

a Estudios que usaron muestras de personas sanas (S) y sujetos con otras infecciones (OI), a) Bhatnagar 25 S y 20 OI, b) Pongsiri 165 S y 65 OI, c) Chen 30 S y 106 OI, d) Yathi 54 S y 35 OI.

b Todos corresponden a OI.

## DISCUSIÓN

En esta revisión se hallaron resultados favorables para ELISA IgM y qPCR en 1 288 sujetos con chikungunya, 1 108 sanos y 394 con otras infecciones, lo que evidencia una mayor posibilidad de inferencia o extrapolación de resultados, un mayor poder estadístico y precisión en los parámetros de utilidad diagnóstica. Esto pone de manifiesto la relevancia de esta modalidad de investigación para la salud pública basada en la evidencia, particularmente en el marco de los programasde tamización de una infección con alta ocurrencia en América.

La mayoría de estudios proceden de Asia, lo que coindice con el origen y diseminación de esta infección inicialmente limitada a África y el sur de Asia (4,15). Sin embargo, desde el brote de 2005 en la isla de la Reunión se ha reportado más un de un millón de casos sospechosos y alrededor de 30 000 confirmados en América, lo que constituye un problema de salud pública en el continente que amerita mayores esfuerzos investigativos, particularmente en evaluación de pruebas diagnósticas como base para brindar tratamiento, ralentizar la transmisión, optimizar el uso de recursos sanitarios y consolidar programas de prevención (5,6,26).

***Fuente:*** elaboración propia a partir de los resultados de la búsqueda

Esta investigación evidencia la importancia y novedad de los metanálisis de pruebas diagnósticas aplicables en diferentes contextos, particularmente ELISA IgM y qPCR que presentaron excelente utilidad para chikungunya, a partir de la combinación de estudios con una alta heterogeneidad en la validez diagnóstica. Frente a estas pruebas se debe precisar que, si bien en la mayoría de infecciones virales es clara su utilidad diagnóstica, en el caso de chikungunya los CDC *(Centers of Disease Control and Prevention)* y la FDA *(Food and Drugs Administration)* unificaron su uso recientemente; sólo en abril de 2017 la FDA autorizó el uso de RT-PCR modificada para mejorar el diagnóstico en personas que cumplen criterios clínicos o epidemiológicos de infección, y en junio de 2016 el uso de emergencia de la prueba MAC-ELISA en laboratorios especializados (certificados para realizar pruebas de alta complejidad) de Estados Unidos (27).

El diagnóstico de esta infección generalmente se hace con base en la sintomatología, la cual es similar a la de dengue y deriva en errores de detección, al tiempo que evidencia la necesidad de identificar pruebas con buena utilidad diagnóstica para confirmar la sospecha clínica. Aunque el aislamiento viral es el estándar de referencia, esta técnica puede detectar el virus en los tres primeros días de la enfermedad y requiere laboratorios especializados, lo que afecta su factibilidad para programas de prevención y ha derivado en una mayor uso de pruebas basadas en la detección ácidos nucleicos, anticuerpos neutralizantes y detección de IgM específica (26,28)

La ELISA IgM presentó excelentes valores en CPP, CPN y ORD, aunque se debe tener presente la variabilidad en especificidad y sensibilidad, atribuible al tiempo de toma de la muestra y la reactividad cruzada(26); por ejemplo en los estudios metanalizados la especificidad osciló entre 81,3% y 100% (9), lo que resulta similar a los estudios de Prat y cols.(29) y Yap et al (30) quienes reportan especificidades del 88% al 100%; mientras que la sensibilidad fue 97,1% lo que difiere de lo reportado por el Instituto de Investigación Biomédica de las Fuerzas Armadas de Francia cuyo resultado estuvo entre 79% y 85% (29) y un estudio del Instituto Robert Koch en 30 laboratorios donde se obtuvo un diagnóstico correcto en el 50,7% de los casos evaluados con esta prueba(31).

En adición a lo anterior, otros estudios con ELISA IgM muestran diferencias cercanas al 20% en la sensibilidad hallada en diferentes poblaciones. Específicamente la mutación A226V del CHIKV ha sido asociada a una mayor capacidad de replicación en el vector Aedes albopictus implicado en el brote de 2005 y en la rápida dispersión del virus en América (32), con un estudio que reporta una disminución de la sensibilidad de un 94% al 75% cuando se evalúa en muestras del brote que se generó por el virus mutado (30).

Los estudios basados en detección de ácidos nucleicos del virus mostraron menor variabilidad en los valores de sensibilidad, especificidad, CPP, CPN y ORD, al tiempo que puede clasificarse como excelente ayuda diagnóstica según los parámetros de CPP mayor a 10, CPN menor a 0,1 y ORD mayor a 100; lo que implica mayor posibilidad de generalización de los resultados, siempre y cuando se tenga presente que en este metanálisis los genes del virus más usados fueron los de envoltura (E) (4,11,14,16,17,20,21,25)y las proteínas no estructurales del virus (nsP) (12,18-19). Por su parte, los valores de sensibilidad y especificidad de la inmunocromatografia son similares a lo encontrado en estudios previos que reportan baja utilidad para el diagnóstico de CHIKV, con sensibilidades del 12,2% al 30%; incluso Prat y cols. (29) indican que esta prueba no debe ser utilizadas en situaciones clínicas, independiente del origen geográfico de la infección (29,30)

**CUADRO 2.** Utilidad de ELISA IgM, qPCR e Inmunocromatografia IgM en el diagnóstico de chikungunya

	Sensibilidad (IC95%)	Especificidad (IC95%)	CPP (IC95%)	CPN (IC95%)	ORD (IC95%)
ELISA IgM				
Estudio				
Shukla J	95,2 ^(88,3-98,7)^	99,1 ^(95,3-100)^	111,4 ^(15,8-784,9)^	0,05 ^(0,02-0,12)^	2 320 ^(254-21 143)^
Bhatnagar S	100 ^(92,1-100)^	100 ^(92,1-100)^	91,0 ^(5,8-1 433)^	0,01^(0,00-0,17)^	8 281 ^(161-426 402)^
Matheus S	100 ^(93,6-100)^	93,8 ^(85,0-98,3)^	14,5 ^(6,0-35,5)^	0,00 ^(0,00-0,15)^	1 544 ^(81-29 329)^
Goh L	100 ^(94,0-100)^	94,7 ^(74,0-99,9)^	13,2 ^(2,8-61,6)^	0,01 ^(0,00-0,14)^	1 492 ^(58-38 207)^
Wasonga C	91,1 ^(80,4-97,0)^	96,7 ^(90,8-99,3)^	27,9 ^(9,2-85,3)^	0,09 ^(0,04-0,21)^	303 ^(69-1 319)^
Wasonga C	97,6 ^(87,1-99,9)^	81,3 ^(72,6-88,2)^	5,2 ^(3,5-7,8)^	0,03 ^(0,00-0,21)^	174 ^(23-1 342)^
Metanálisis				
Combinada	97,1 ^(94,7-98,6)^	93,5 ^(90,8-95,6)^	21,1 ^(6,0-74,1)^	0,04 ^(0,02-0,09)^	687 ^(238-1 985)^
Heterogeneidad ^X2^	15,1 p=0,010	35,5 p<0,001	38,5 p<0,001	7,2 p=0,209	6,10 p=0,297
Inconsistency ^I2^	66,9 %	85,9 %	87,0 %	30,1 %	18,0 %
qPCR				
Estudio				
Edwards C	100 ^(81,5-100)^	86,5 ^(71,2-95,5)^	6,7 ^(3,1-14,6)^	0,03 ^(0,00-0,48)^	219 ^(11-4 181)^
Santhosh S	100 ^(92,1-100)^	62,5 ^(35,4-84,8)^	2,6 ^(1,4-4,7)^	0,02 ^(0,00-0,28)^	147 ^(8-2 819)^
Kumar Dash P	100 ^(84,6-100)^	100 ^(83,2-100)^	41,1 ^(2,7-635,9)^	0,07 ^(0,03-0,17)^	1845 ^(35-97 304)^
Pongsiri P	100 ^(94,0-100)^	94,7 ^(90,9-97,2)^	18,1 ^(10,5-31,0)^	0,05 ^(0,01-0,24)^	2086 ^(122-35 742)^
Andriamandimby S	93,2 ^(84,7-97,7)^	96,3 ^(90,8-99,0)^	25,2 ^(9,6-65,9)^	0,07 ^(0,01-0,31)^	354 ^(92-1 364)^
Chiam C	96,7 ^(82,8-99,9)^	100 ^(83,2-100)^	40,0 ^(2,6-618,8)^	0,05 ^(0,01-0,18)^	806 ^(31-20 793)^
Chen H	95,5 ^(77,2-99,9)^	100 ^(83,2-100)^	39,3 ^(2,5-608,5)^	0,07 ^(0,01-0,31)^	588 ^(23-15 267)^
Chen H	95,5 ^(84,5-99,4)^	98,6 ^(94,9-99,8)^	65,9 ^(16,6-261,1)^	0,05 ^(0,01-0,18)^	1428 ^(195-10 449)^
Metanálisis				
Combinada	97,1 ^(94,6-98,7)^	95,1 ^(93,0-96,7)^	16,4 ^(6,4-42,0)^	0,05 ^(0,03-0,09)^	560 ^(241-1 302)^
Heterogeneidad ^X2^	12,0 p=0,099	31,4 p<0,001	43,3 p<0,001	4,0 p=0,778	3,7 p=0,814
Inconsistency ^I2^	41,9 %	77,7 %	83,8 %	0,0 %	0,0 %
Inmunocromatografía				
Estudio				
Arya S	100 ^(47,8-100)^	90,5 ^(82,8-95,6)^	9,3 ^(4,8-17,7)^	0,09 ^(0,01-1,31)^	100 ^(5-1 955)^
Kosasih H	67,7 ^(59,0-75,5)^	89,2 ^(79,8-95,2)^	6,3 ^(3,2-12,2)^	0,36 ^(0,28-0,47)^	17 ^(8-39)^
Kosasih H	20,5 ^(13,9-28,3)^	100 ^(95,1-100)^	31,0 ^(1,9-501,2)^	0,80 ^(0,73-0,87)^	39 ^(2-647)^
Okabayasi T	89,5 ^(80,3-95,3)^	94,4 ^(81,3-99,3)^	16,1 ^(4,2-62,1)^	0,11 ^(0,06-0,22)^	144 ^(29-718)^
Metanálisis				
Combinada	54,9 ^(49,5-60,2)^	93,2 ^(89,6-95,9)^	8,5 ^(5,5-13,2)^	0,28 ^(0,08-0,94)^	45 ^(13-164)^
Heterogeneidad ^X2^	124 p<0,001	13,1 p=0,004	2,7 p=0,432	153 p<=0,00	6,1 p=0,106
Inconsistency ^I2^	97,6 %	77,1 %	0,0 %	98,0 %	50,9 %

***Fuente:*** elaboración propia a partir de los resultados presentados.

CPP: Cociente de Probabilidad Positivo; CPN: Cociente de Probabilidad Negativo; ORD: Razón de Odds Diagnóstica; se adicionó 0,5 a las celdas de estudios con cero.

Es oportuno precisar que la mayoría de estudios incluidos en esta revisión sólo reportan sensibilidad y especificidad, por lo que los demás parámetros fueron estimados como valor adicional de este metanálisis. En relación con los coeficientes de probabilidad vale decir que estos permiten establecer cuanto más probable es un resultado, positivo o negativo, dependiendo de la presencia o la ausencia de la infección, sin ser afectados por la prevalencia. Las curvas ROC permiten determinar el punto en el que se alcanza la mayor sensibilidad y especificidad de la prueba, es decir el punto donde se presenta la mejor capacidad discriminativa entre la presencia o ausencia de la condición, lo que constituye un índice de validez, que en este metanálisis evidenció mejor resultado para ELISA IgM (33,34)

Esta revisión presenta limitaciones como la poca cantidad de artículos para algunas de las metodologías evaluadas como es el caso de la inmunocromatografía, cuyos valores de sensibilidad prestaron alta variabilidad, un bajo poder estadístico para las comparaciones con las demás pruebas y baja potencia de los estadísticos que evalúan los sesgos de publicación; esto impide concluir sobre la validez de esta prueba, al tiempo que se evidencia la necesidad de estudios posteriores que respalden su uso en programas de tamización. A esto se suma la ausencia de estudios que evaluaran pruebas como la detección de antígenos, el aislamiento viral o la inmunofluorescencia que son pruebas usadas en el diagnóstico de esta arbovirosis. Una limitación mayor radica en la imposibilidad de identificar las causas de heterogeneidad en los parámetros de utilidad diagnóstica debido a la baja exhaustividad de los estudios individuales en el reporte de variables como la edad, el sexo, el momento de toma, signos y síntomas, entre otras.

***Fuente:***elaboración propia a partir de los resultados presentados.

No obstante estas limitaciones, se destaca como fortalezas del estudio la identificación de las pruebas evaluadas para el diagnóstico de la infección, la generación de evidencia sobre la utilidad diagnóstica de la ELISA IgM y la q PCR con una alta validez externa y posibilidades de extrapolación a diferentes países, así como países con necesidad de mayor investigación en este tema. Además, las revisiones sistemáticas permiten conocer de manera sintetizada la información disponible, aumentar el tamaño de muestra, mejorar la potencia estadística y generar conclusiones que no podrían derivarse de los estudios individuales (35, 36)

Se concluye que las pruebas con mejor utilidad diagnóstica para chikungunya son la ELISA IgM y qPCR, lo que resulta de gran relevancia para programas de prevención de esta infección, para la orientación de decisiones clínicas, así como para el desarrollo de investigaciones posteriores que permitan mejorar el grado de recomendación de los hallazgos de este estudio, mejorar el diagnóstico precoz y brindar un tratamiento oportuno.

### Declaración

Las opiniones expresadas en este manuscrito son responsabilidad del autor y no reflejan necesariamente los criterios ni la política de la RPSP/PAJPH y/o de la OPS
